# More Consistently Altered Connectivity Patterns for Cerebellum and Medial Temporal Lobes than for Amygdala and Striatum in Schizophrenia

**DOI:** 10.3389/fnhum.2016.00055

**Published:** 2016-02-17

**Authors:** Henning Peters, Junming Shao, Martin Scherr, Dirk Schwerthöffer, Claus Zimmer, Hans Förstl, Josef Bäuml, Afra Wohlschläger, Valentin Riedl, Kathrin Koch, Christian Sorg

**Affiliations:** ^1^Department of Psychiatry, Technische Universität MünchenMünchen, Germany; ^2^TUM-Neuroimaging Center, Klinikum Rechts der Isar, Technische Universität MünchenMünchen, Germany; ^3^Klinik für Psychiatrie und Psychotherapie, Klinikum der Universität MünchenMünchen, Germany; ^4^School of Computer Science and Engineering, University of Electronic Science and Technology of ChinaChengdu, China; ^5^Big Data Research Center, University of Electronic Science and Technology of ChinaChengdu, China; ^6^Center for Information in BioMedicine, University of Electronic Science and Technology of ChinaChengdu, China; ^7^Department of Neuroradiology, Technische Universität MünchenMünchen, Germany; ^8^Department of Nuclear Medicine, Technische Universität MünchenMünchen, Germany

**Keywords:** fMRI, support vector machine, schizophrenia, functional connectivity (FC), multivariate pattern analysis, subcortical

## Abstract

**Background**: Brain architecture can be divided into a cortico-thalamic system and modulatory “subcortical-cerebellar” systems containing key structures such as striatum, medial temporal lobes (MTLs), amygdala, and cerebellum. Subcortical-cerebellar systems are known to be altered in schizophrenia. In particular, intrinsic functional brain connectivity (iFC) between these systems has been consistently demonstrated in patients. While altered connectivity is known for each subcortical-cerebellar system separately, it is unknown whether subcortical-cerebellar systems’ connectivity patterns with the cortico-thalamic system are comparably altered across systems, i.e., if separate subcortical-cerebellar systems’ connectivity patterns are consistent across patients.

**Methods**: To investigate this question, 18 patients with schizophrenia (3 unmedicated, 15 medicated with atypical antipsychotics) and 18 healthy controls were assessed by resting-state functional magnetic resonance imaging (fMRI). Independent component analysis of fMRI data revealed cortical intrinsic brain networks (NWs) with time courses representing proxies for cortico-thalamic system activity. Subcortical-cerebellar systems’ activity was represented by fMRI-based time courses of selected regions-of-interest (ROIs; i.e., striatum, MTL, amygdala, cerebellum). Correlation analysis among ROI- and NWs-time courses yielded individual connectivity matrices [i.e., connectivity between NW and ROIs (allROIs-NW, separateROI-NW), only NWs (NWs-NWs), and only ROIs (allROIs-allROIs)] as main outcome measures, which were classified by support-vector-machine-based (SVM) leave-one-out cross-validation. Differences in classification accuracy were statistically evaluated for consistency across subjects and systems.

**Results**: Correlation matrices based on allROIs-NWs yielded 91% classification accuracy, which was significantly superior to allROIs-allROIs and NWs-NWs (56 and 74%, respectively). Considering separate subcortical-cerebellar systems, cerebellum-NWs and MTL-NWs reached highest accuracy values with 91 and 85%, respectively, while those of striatum-NW and amygdala-NW were significantly lower with about 65% classification accuracy.

**Conclusion**: Results provide initial evidence for differential consistency of altered intrinsic connectivity patterns between subcortical-cerebellar systems and the cortico-thalamic system. Data suggest that differential dysconnectivity patterns between subcortical-cerebellar and cortical systems might reflect different disease states or patient subgroups.

## Introduction

Widespread brain dysconnectivity is an essential element in the pathophysiology of schizophrenia (Stephan et al., 2009). The cortico-thalamic system is particularly affected by aberrant functional and structural connectivity (Flynn et al., 2003; Begré and Koenig, 2008; Lynall et al., 2010; van den Heuvel et al., 2010; Welsh et al., 2010; Woodward et al., 2012; Palaniyappan et al., 2013; Wagner et al., 2013, 2015; Manoliu et al., 2014). For example, the coherence of ongoing, slowly fluctuating neural activity (<0.1 Hz), which reflects a basic form of brain organization called intrinsic functional connectivity (iFC; Fox and Raichle, 2007), is consistently changed in patients relative to healthy controls for both cortico-thalamic (Welsh et al., 2010; Woodward et al., 2012) and cortico-cortical connectivity (Lynall et al., 2010; Palaniyappan et al., 2013; Manoliu et al., 2014; van den Heuvel and Fornito, 2014). One should note that, rather than overall reduced or increased connectivity, alterations reflect rather complex reorganization of network interaction.

Beyond the cortico-thalamic system, several other non-cortical/non-thalamic brain systems are involved in schizophrenia. For example, the striatum shows increased pre-synaptic dopamine concentrations that are crucial for schizophrenia’s psychotic symptoms (Howes et al., 2012) and increased activity is linked with symptom dimensions, for putamen with positive symptoms during psychosis, for ventral striatum with negative symptoms during remission (Sorg et al., 2013). Moreover, substantial alterations within the medial temporal lobes (MTLs) include altered microstructure, transmitters, perfusion, and activity, particularly in the hippocampus (Tamminga et al., 2010). In more detail, structural deficits were recently attributed to hippocampal subfields in large samples of schizophrenia patients (Mathew et al., 2014; Arnold et al., 2015; Haukvik et al., 2015) and were associated with decreased default-mode-network suppression in healthy subjects at genetic risk (Seidman et al., 2014). Patients’ memory deficits, particularly with regard to relational information (vs. item-specific), have been linked with diminished hippocampal activation (Ragland et al., 2015). Furthermore, abnormalities in amygdala and cerebellum, ranging from molecular to microcircuit alterations, are consistent features of schizophrenia and are linked with emotional and cognitive deficits, respectively (Andreasen and Pierson, 2008; Benes, 2010; Anticevic et al., 2012; Mier et al., 2014; Millan et al., 2014; Vai et al., 2015). In more detail, amygdala alterations are heterogeneous and appear to differ depending on the clinical syndrome and disorder state (Rasetti et al., 2009). For example, amygdala hyperactivity has been shown to relate to paranoid ideation in schizophrenia, suggesting symptom-based subgroups (Pinkham et al., 2015). Findings across subjects at high-risk, early and chronic states of schizophrenia, suggest differentially affected amygdala iFC from increased to reduced levels, also reflecting symptom severity (Anticevic et al., 2014; Mukherjee et al., 2014). Considering altered cerebellar functional connectivity, mainly reduced connectivity has been reported consistently across different tasks and disorder states (Lungu et al., 2013). Interestingly, the cerebellum has been identified as one of the key regions of hypoconnectivity with the thalamus related to conversion of subjects at ultra high risk to psychosis (Anticevic et al., 2015).

Intriguingly, all these brain systems—striatum, MTL, amygdala, and cerebellum—are massively connected with the cortico-thalamic system (Swanson, 2003). They are assumed to play key modulatory roles in cortico-thalamic processes (Buzsaki, 2006) and beyond structural aberrations (Bois et al., 2015; van Erp et al., 2015), several studies demonstrated their aberrant iFC with the cortico-thalamic system in patients with schizophrenia (Collin et al., 2011; Anticevic et al., 2012; Fornito et al., 2013; Kraguljac et al., 2014; Peters et al., in press). We use the term “subcortical-cerebellar system” to refer to these systems. We are aware that it is a matter of debate to separate these systems from the cortico-thalamic system; for example, the striatum is massively integrated in cortico-basal ganglia-thalamo-cortical loops. However, differences in anatomy and connectivity, physiology and function, as well as their distinct role in schizophrenia pathophysiology suggest that treating these systems separately from thalamo-cortical connectivity is plausible (Buzsaki, 2006; Andreasen and Pierson, 2008; Benes, 2010; Tamminga et al., 2010; Howes et al., 2012). While previous studies investigated each “subcortical-cerebellar” system and its connectivity separately, it is unknown how the whole pattern of connectivity changes between “subcortical-cerebellar” systems and the cortico-thalamic system is configured in schizophrenia, particularly whether abnormal connectivity is consistent across patients for different systems. This might be an important question, since graded patterns of aberrant connectivity across subcortical-cerebellar systems might vary across disease states and patient subgroups. To obtain initial evidence for this question, the current study investigates connectivity patterns in the cortico-thalamic system across mentioned subcortical-cerebellar systems, and—most importantly—between these two types of systems in psychotic patients with schizophrenia to evaluate the inter-individual consistency of potential changes across systems.

**Figure 1:**
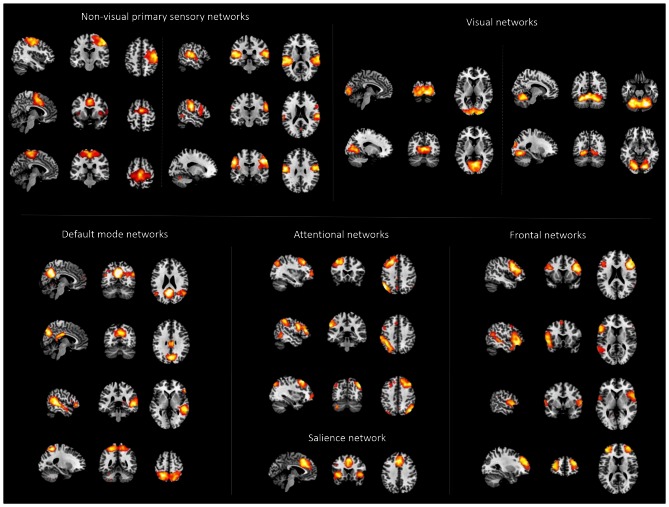
**Cortical networks.** Displayed are spatial maps of cortical networks (NWs) derived from independent component analysis (ICA) of patients’ and healthy controls’ resting state fMRI data, representing the cortico-thalamic sub-systems. The 22 NWs shown were selected following an automated spatial multiple-regression on reference-templates provided by Allen et al. (2011).

**Figure 2:**
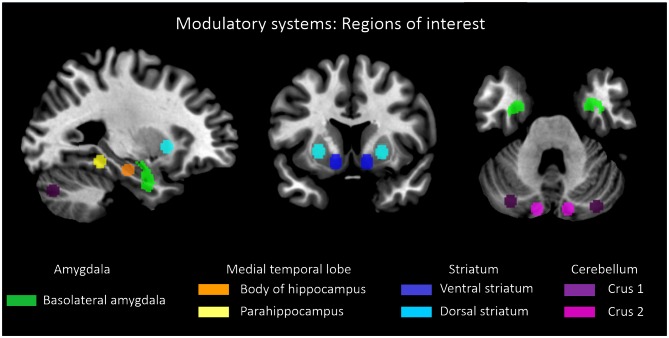
**Subcortical-cerebellar systems.** Displayed are regions of interest (ROI) based on coordinates derived from previous studies in case of striatum, medial temporal lobe and cerebellum (Kahn et al., 2008; Etkin et al., 2009; Krienen and Buckner, 2009; Peters et al., in press). Definition of amygdala ROI was derived from Anatomy toolbox for SPM (http://www.fz-juelich.de/inb/inb-3/spm_anatomy_toolbox).

To reach the study’s objective, the following steps have been performed (for an overview, see Figure S1): (i) We assessed slowly fluctuating ongoing brain activity (Leopold and Maier, 2012) in patients with schizophrenia and healthy controls with resting-state functional MRI (rs-fMRI). (ii) Ongoing brain activity is organized in intrinsic brain networks (NWs) characterized by specific iFC patterns (Fox and Raichle, 2007). We used intrinsic networks, which cover preferentially the cerebral cortex, as proxy for cortico-thalamic sub-systems (Figure 1). Corresponding time courses, reflecting networks’ activity (NWs) were used for further analysis. (iii) Concerning subcortical-cerebellar systems, we focused on striatum, amygdala, MTL, and cerebellum (Figure 2). For each system, we chose typical sub-regions, which have been used previously to study systems’ iFC (details in the method part). For these regions-of-interest, we extracted representative rs-fMRI signal time courses (ROIs). (iv) Then, we calculated iFC connectivity matrices (i.e., Pearson correlation of pairs of signal time courses of NWs or ROIs, respectively) for combinations of allROIs-NWs, NWs-NWs, allROIs-allROIs as well as separateROI-NWs for each subject. (v) Finally, we used multivariate pattern classification (i.e., support vector machine) and leave-one-out cross validation to estimate group differences and particularly inter-individual consistency of iFC patterns for different subcortical-cerebellar systems.

We hypothesized that: 1. Connectivity matrices based on allROIs-NWs reflect more consistent connectivity patterns within groups of patients and healthy controls than allROIs-allROIs and NWs-NWs connectivity matrices. Therefore, they classify subjects more accurately. 2. Separate-ROI-NWs connectivity matrices describe differentially consistent connectivity patterns. Therefore, separate ROIs classify subjects with different accuracy.

## Materials and Methods

Eighteen healthy controls and Eighteen patients participated in the study (Table 1). All participants had been investigated in a previous study (Manoliu et al., 2014), which addressed cortical iFC changes for three selected intrinsic networks i.e., default mode, salience, and central executive network; here, we focus on a larger number of cortical networks and their iFC with subcortical-cerebellar systems. Written informed consent in accordance with the Human Research Committee guidelines of the Klinikum Rechts der Isar, Technische Universität München was obtained from all participants. Patients were recruited from the Department of Psychiatry, Klinikum Rechts der Isar, TU München, controls by word-of-mouth advertising. Participants’ examination included medical history, psychiatric interview, psychometric assessment, and additionally blood tests for patients. Psychiatric diagnoses relied on the Diagnostic and Statistical Manual of Mental Disorders (DSM-IV). To assess psychiatric diagnoses, the Structured Clinical Interview for DSM-IV (SCID-I) was used (Spitzer et al., 1992). The global level of social, occupational, and psychological functioning was measured with the Global Assessment of Functioning Scale (GAF; Spitzer et al., 1992). For rating severity of clinical symptoms on the day of scanning, the Positive and Negative Syndrome Scale (PANSS) was applied (Kay et al., 1987). Clinical-psychometric assessment was completed by psychiatrists DS and MS who have been professionally trained for SCID and PANSS-based interviews with inter-rater reliability of more than 95%. Inclusion criteria for the study were diagnosis of schizophrenia and age between 18 and 60 years. Exclusion criteria were current or past neurological or internal systemic disorder, current depressive or manic episode, substance abuse (except for nicotine), schizoaffective disorder, and cerebral pathology in MRI.

**Table 1:** **Demographic and psychometric data**.

	SP (*n* = 18)	HC (*n* = 18)	SP vs. HC	
Measure	Mean (SD)	Mean (SD)	*T*-score	*p*-value
Age	35.33 (12.49)	34.10 (13.4)	0.318	0.75
Sex (m/f)	9/9	9/9		
PANSS-Total	76.44 (18.45)	30.10 (0.66)	11.23	<0.001*
PANSS-Positive	18.06 (5.74)	6.95 (0.23)	8.57	<0.001*
PANSS-Negative	19.94 (8.11)	6.90 (0.44)	7.08	<0.001*
GAF	41.50 (11.55)	99.50 (1.12)	−22.46	<0.001*
CPZ	466.72 (440.49)

Two-sample t-tests for age and psychometric tests; *significant for p < 0.05. Abbreviations: SP, patients with schizophrenia during acute psychosis; HC, healthy control group; PANSS, Positive and Negative Syndrome Scale; GAF, Global Assessment of Functioning Scale; CPZ, chlorpromazine equivalent dose.

All patients included were diagnosed with paranoid schizophrenia during acute psychosis as indicated by clinical exacerbation and increased positive symptom scores on the PANSS (Table 1). Seven out of eighteen had significant hallucinations (PANSS P3 ≥ 3), 15 delusions (P1 ≥ 3). The mean duration of illness was 7.15 years (SD = 6.89 years), the mean number of hospital stays was 2.98 (SD = 2.48). Concerning medication, three patients were free of any antipsychotic medication, the others received mono- or dual therapy with atypical antipsychotic medication including Amisulpride (*n* = 2), Olanzapine (*n* = 11), Clozapine (*n* = 4), Quetiapine (*n* = 2), Ziprasidone (*n* = 1), Risperidone (*n* = 5), Aripiprazole (*n* = 2), Paliperidone (*n* = 3; cp. Table S2 for individual medication protocols and dosage; see Table S1 for mean chlorpromazine equivalent dose). All healthy subjects were free of psychotropic medication and any neurological and psychiatric disorder, current and in history.

## Data Acquisition

All participants underwent 10 min of rs-fMRI with the instruction to keep their eyes closed and not to fall asleep. We verified that subjects stayed awake by interrogating via intercom immediately after the rs-fMRI scan. Before and after scanning, a medical examination of patients validated their stable condition and investigated whether they had feelings of odd situations during the scanning. No patient dropped out during the scanning session.

MRI was carried out on a 3 T whole body scanner (Achieva, Philips Healthcare). FMRI was based on gradient echo EPI sequence (TE = 35 ms, TR = 2000 ms, flip angle = 82°, FoV = 220 × 220 mm^2^, matrix = 80 × 80, 32 slices, slice thickness = 4 mm, and 0 mm interslice gap; 300 volumes). T1-weighted anatomical MRI was based on magnetization-prepared rapid acquisition gradient echo sequence (TE = 4 ms, TR = 9 ms, TI = 100 ms, flip angle = 5°, FoV = 240 × 240 mm^2^, matrix = 240 × 240, 170 slices, voxel size = 1 × 1 × 1 mm^3^).

## Functional MRI Data Preprocessing and Analysis

### Preprocessing

For each participant, the first three rs-fMRI scans were discarded due to magnetization effects. SPM8 (Wellcome Department of Cognitive Neurology, London) was used for motion correction, spatial normalization into the stereotactic space of the Montreal Neurological Institute (MNI) and spatial smoothing with an 8 mm × 8 mm × 8 mm Gaussian kernel. To ensure data quality, particularly concerning motion-induced artifacts, temporal signal-to-noise ratio (tSNR) and point-to-point head motion were estimated for each subject (Murphy et al., 2007; Van Dijk et al., 2012). Point-to-point motion was defined as the absolute displacement of each brain volume compared to its previous volume. Moreover, root mean square (RMS) of the translational head movement parameters was calculated for each subject. Excessive head motion (cumulative motion translation >3 mm and mean point-to-point translation or rotation >0.15 mm or 0.1°) was applied as exclusion criterion. None of the participants had to be excluded. Two-sample *t*-tests yielded no significant differences between groups regarding mean point-to-point translation or rotation of any direction (*p* > 0.18), RMS (*p* > 0.25), or tSNR (*p* > 0.35).

### Subcortical-Cerebellar Systems: ROIs and Preprocessing

For each subcortical-cerebellar system, representative ROIs were defined based on previous studies, which investigated systems’ cortical iFC (Kahn et al., 2008; Etkin et al., 2009; Krienen and Buckner, 2009; Peters et al., in press; Figure 2). For the amygdala, left and right basolateral amygdala ROI were derived from the Anatomy toolbox for SPM^1^ following Etkin et al. (2009) and converted to corresponding ROIs via Marsbar^2^. For other subcortical-cerebellar systems, center coordinates in striatum, cerebellum, and MTL, respectively, were derived from the literature and spherical ROIs (6 mm radius) were created via Marsbar: left and right ventral and dorsal striatum following Peters et al. (in press) with MNI coordinates [*XYZ*]: (±12, 9, −9) and (±24, 12, 0), left and right Crus 1 and 2 following Krienen and Buckner (2009) with MNI coordinates [*XYZ*]: (±12.0, −80.0, −24.0) and (±22.0, −86.0, −40.0), left and right hippocampus and parahippocampus ROIs following Kahn et al. (2008) with MNI coordinates [*XYZ*]: (±24.0, −18.0, −18.0) and (±26.0, −40.0, −12.0).

Using in-house scripts employing SPM 8 and matlab^3^ routines, for each subject and ROI, fMRI-time courses were extracted, Butterworth bandpass-filtered for the frequency range from 0.009 to 0.08 Hz, and reduced to ROI-representative time courses by singular value decomposition. To account for partial volume and movement effects, linear effects of global gray matter (GM), white matter (WM), cerebrospinal fluid (CSF) fMRI-signal, and six movement parameters were regressed out. We included the global GM signal as a nuisance regressor since it is thought to reflect a combination of physiological processes (such as cardiac and respiratory fluctuations) and scanner drift. To extract the nuisance covariate time series for GM, WM and CSF, each individual’s high-resolution T1-weighted structural image was segmented. Mean images of the study sample’s T1-segmentation were used to create ROIs for the extraction of GM, WM, and CSF nuisance signals.

### Cortico-Thalamic Networks: NW Identification and Selection

NWs were identified by ICA of pre-processed fMRI data within an identical framework as defined by Allen et al. (2011) and as applied previously (Manoliu et al., 2014). Briefly, selection of the optimal ICA model-order to analyze rs-fMRI data is subject of ongoing debate. However, it has been demonstrated that a model-order around 70 components may represent an optimal level to detect between-group differences and to avoid false positive results. Therefore, we followed the approach of Allen et al. (2011), who used ICA model order of 75 components. Further reasons to follow Allen’s approach were both greater comparability of results across studies and most importantly, reduction of subjective bias for network selection by using Allen’s templates to identify networks-of-interest. Preprocessed data were decomposed into 75 spatially independent components within a group-ICA framework based on the infomax-algorithm as implemented in the GIFT-software^4^. FMRI data were concatenated and reduced by two-step principal component analysis, followed by independent component estimation with the infomax-algorithm. We subsequently ran 40 ICA (ICASSO) to ensure stability of the estimated components. This resulted in a set of average group components, which are then back-reconstructed into single subject space. Each back-reconstructed component consists of a spatial z-map reflecting the component’s functional connectivity pattern across space and an associated time course reflecting component’s activity across time.

To select ICs which covered mainly the cortico-thalamic system, we chose templates as provided by Allen et al. (2011), i.e., t-maps of 28 components that reflect canonical intrinsic networks. We chose components mainly covering the cerebral cortex (22 of 28 maps, see Supplementary Material) to reflect networks-of-interest in an automated and objective way, and performed multiple spatial regression analyses of our 75 independent components’ spatial maps on these templates. Components of highest correlation coefficient with the templates were selected, resulting in 22 ICs of interest. In the end, this approach yielded a component’s z-map and time course for each subject and cortical intrinsic network, which reflect network’s coherent activity. Time courses of NW were used as proxies for cortico-thalamic system activity for further analysis.

### Classification Procedure

To generate individual feature vectors as input parameter for pattern classification via SVM, respective Pearson-correlations of NWs’ and ROIs’ time courses and subsequent Fisher’s r-to-z transformation were performed for each subject (allROIs-NWs) and represented as typical correlation matrices. Correlation matrices for NWs-NWs and allROIs-allROIs connectivity were analogously created. Besides a global analysis including all ROIs and NWs as input for classification, also separateROIs-NWs correlation matrices were analyzed, including only one of the four subcortical-cerebellar system-ROIs at a time (i.e., cerebellum, amygdala, striatum, or MTL), thus facilitating comparison of classification accuracy obtained by each of the subcortical-cerebellar systems’ component.

The resulting correlation matrices of the two groups (feature vectors) were used for pattern recognition based classification via the WEKA toolbox^5^. Specifically, a support-vector machine based algorithm (SVM) was applied together with leave-one-out cross validation. The basic idea of SVM procedures (Vapnik, 1999) is to construct a separating hyperplane between multi-dimensional training instances (i.e., correlation matrices) of both classes (i.e., groups). Among all possible hyperplanes, that one with the maximum margin between classes is selected and subsequently applied to classify test instances. In order to evaluate classification effectiveness, SVM procedure has been realized within a leave-one-out validation framework, in which in each round of classification one subject is used as test instance while the others represent training instances. The specific algorithm implemented in WEKA applies sequential minimal optimization and a linear kernel (Platt, 1999). Measures-of-interest for SVM-based classification were classification accuracy, sensitivity and specificity, which together reflect the power of the particular feature (i.e., NWs-NWs, allROIs-allROIs, allROIs-NWs, and separateROIs-NMs) to discriminate groups. These measures reflect how consistent patients/controls are correctly classified due to the corresponding iFC pattern. We intended to examine different subcortical-cerebellar systems for such consistency of connectivity pattern changes. In addition, statistical significance of differences in classification measures was determined by reading out the individual classification results (i.e., correct or misclassified) and pairwise identification of diverging classification accuracy for pairs of features between NWs-NWs, allROIs-allROIs, allROIs-NWs, and separateROIs-NWs (Japkowicz and Shah, 2011). In more detail, let (X_1_,Y_1_), (X_2_,Y_2_), …, (X_35_,Y_35_) indicate the individual classification results, where X_i_ = 0 or Y_i_ = 0 (misclassified), X_i_ = 1 or Y_i_ = 1 (correct). We counted the number of pairs X_i_ > Y_i_. Let W be the number of pairs (X_i_ > Y_i_), N is the number of pairs that X_i_ and Y_i_ are different, then W follows a binomial distribution W ~ B(N, 0.5). The exact *p*-value was obtained by checking binomial distribution.

## Results

### Typical Cortical Intrinsic Networks have been Identified in Patients and Controls

ICA analysis of rs-fMRI data from patients and controls identified 22 intrinsic networks, which cover mainly the cerebral cortex (Figure 1; for more details, Figures S2–6). Networks were consistent across groups, and matched with previously defined networks of an identical methodological approach in 603 healthy controls (Allen et al., 2011).

### Superior Classification Accuracy for allROIs-NWs than allROIs-allROIs or NWs-NWs

When separating individual patients from healthy controls via SVM classification accuracy for iFC patterns between all subcortical-cerebellar systems’ ROIs and cortical NWs (allROIs-NWs) was 91% with high sensitivity and specificity levels of about 90% (Table 2). Classification accuracies for iFC patterns among subcortical-cerebellar systems allROIs-allROIs and cortical networks NWs-NWs, were significantly lower with 56 and 73%, respectively (Tables 2, 4).

**Table 2:** **Classification results based on intrinsic functional connectivity (iFC) among subcortical-cerebellar systems and cortical networks, respectively**.

Connectivity matrix	Classification accuracy [%]	Sensitivity	Specificity
allROI-allROI	55.9	0.59	0.53
NW-NW	73.5	0.71	0.77
allROI-NW	91.2	0.88	0.94

*Classification results based on support vector machine algorithm and leave-one-out cross validation. Input to the classifier were connectivity matrices based on Pearson correlations among time courses of region-of-interest (ROI) of subcortical-cerebellar systems and cortical intrinsic networks (NW) of Figures S2–6*.

### Superior Classification Accuracy for MTL-NWs and Cerebellum-NWs than Striatum-NWs and Amygdala-NWs

**Figure 3:**
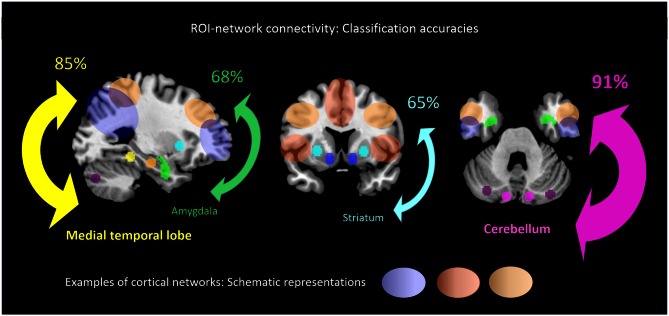
**ROI-network connectivity.** Connectivity matrices between cortical networks (NWs, cp. Figure 1) and different ROI, i.e., cerebellum, medial temporal lobe, amygdala and striatum (cp. Figure 2), served as input features for support-vector-machine-based classification of schizophrenia patients and healthy subjects. Displayed classification accuracies [%] indicate differential discriminatory power of separate ROI-NWs-systems and the degree of inter-subject consistency of ROI-specific connectivity pattern changes in schizophrenia.

When separating individual patients from healthy controls via SVM for different subcortical-cerebellar systems separately, classification accuracies were significantly higher for iFC patterns of cerebellum-NWs (91%, sensitivity 82%, specificity 100%) and MTL-NWs (85%, sensitivity 77%, specificity 94%) than for striatum-NWs (65%, sensitivity 65%, specificity 65%) and amygdala-NW (68%, sensitivity 71%, specificity 65%; Table 3 and S3, Figure 3).

**Table 3:** **Classification results based on intrinsic functional connectivity (iFC) between single subcortical-cerebellar systems and cortical networks**.

Connectivity matrix	Classification accuracy [%]	Sensitivity	Specificity		
Cerebellum-NWs	91.3	0.82	1.00
MTL-NWs	85.3	0.77	0.94
Striatum-NWs	64.7	0.65	0.65
Amygdala-NWs	67.6	0.71	0.65

*Classification results based on support vector machine algorithm and leave-one-out cross validation. Input to the classifier were connectivity matrices based on Pearson correlations between time courses of region-of-interest (ROI) of subcortical-cerebellar systems and cortical intrinsic networks of Figures S2–6*.

**Table 4:** **Statistical evaluation of differences in classification accuracy for different connectivity matrices**.

	allROI-NW	NW-NW	allROI-allROI
allROI-NW	–	0.0439*	0.0002**
NW-NW		–	0.0439*

Distinct connectivity matrices based on Pearson correlations among time courses of regions-of-interest (ROI) of subcortical-cerebellar systems (Figure 1) and cortical intrinsic networks (NW) of Figures S2–6 have been classified by support vector machine with distinct classification accuracy results (see Table 2). Classification accuracies were compared among each other via statistical testing (pairwise binominal testing, details in methods). allROI-NW connectivity yielded significantly highest classification accuracy (*indicates significance at threshold p = 0.05, **p = 0.01). Abbreviations: ROI, Regions of interest; NW, networks.

## Discussion

The current study focused on iFC between different subcortical-cerebellar systems (ROIs) and cortical intrinsic networks (NWs) in patients with schizophrenia. (i) Taking all subcortical-cerebellar systems of interest together, i.e., MTL, cerebellum, striatum, and amygdala (allROIs-NWs), iFC separated patients from controls with an accuracy of about 90%, which was significantly superior in comparison to allROIs-allROIs and NWs-NWs connectivity. This result highlights the role of iFC between subcortical-cerebellar systems and cortical networks in contrast to both cortico-cortical and subcortical-cerebellar-subcortical-cerebellar systems’ connectivity in schizophrenia. (ii) Across subcortical-cerebellar systems, MTL-NWs and cerebellum-NWs connectivity showed highest classification accuracies of about 90%, which were significantly higher in comparison to iFC patterns of striatum-NWs and amygdala-NWs with accuracies of about 65%. This finding provides first evidence for differential across-system consistency of changes in intrinsic connectivity between different subcortical-cerebellar systems and the cortico-thalamic system. Data suggest that the whole pattern of cortical-subcortical-cerebellar system dysconnectivity might be differential for patients, maybe indicating different patient subgroups or disease states.

### iFC Between Subcortical-Cerebellar Systems and Cortical Networks is more Consistently Changed than iFC within Modulatory Systems and Cortical Networks, Respectively

Changes in iFC patterns between all subcortical-cerebellar and cortical networks are highly consistent in psychotic patients with schizophrenia indicated by classification accuracy, sensitivity, and specificity of about 90% (Table 2). Interestingly, such iFC changes are more consistent than those among only subcortical-cerebellar (allROIs-allROIs) or only cortical networks (NWs-NWs), respectively, highlighting the prominent role of subcortical-cerebellar system connectivity into the cortico-thalamic system in schizophrenia (Tables 2, 5). This result is in line with several previous studies, which demonstrated separately impaired iFC between a selected subcortical-cerebellar system and cortical regions (Collin et al., 2011; Anticevic et al., 2012; Fornito et al., 2013; Kraguljac et al., 2014; Wagner et al., 2015; Peters et al., in press). For instance, in a study by Wagner et al. (2015) an abnormal effective connectivity was observed between thalamus, anterior cingulate and dorsolateral prefrontal cortex in patients with schizophrenia compared to healthy controls. Moreover, patients have been found to show a decreased functional connectivity between the putamen with right anterior insula and dorsal prefrontal cortex and the ventral striatum with left anterior insula compared to healthy control subjects (Peters et al., in press). The present result goes beyond these studies by demonstrating that the whole pattern of iFC between several subcortical-cerebellar systems and cortical systems is more consistently aberrant than iFC among subcortical-cerebellar systems or among cortical systems alone.

**Table 5:** **Statistical evaluation of differences in classification accuracy for different connectivity matrices focused on single subcortical-cerebellar systems**.

	CB	MTL	AY	ST
CB	–	0.2188	0.0097*	0.0095*
MTL		–	0.0537	0.0269*
AY			–	0.2256

Distinct connectivity matrices based on Pearson correlations among time courses of region-of-interest of subcortical-cerebellar systems and cortical intrinsic networks of Figures S2–6 have been classified by support vector machine with distinct classification accuracy results (see Table 3). Classification accuracies were compared among each other via statistical testing (pairwise binominal testing, details in methods). CB-network and MTL-network connectivity yielded significantly highest classification accuracy (*indicates significance at threshold p = 0.05, **p = 0.01). Abbreviations: CB, cerebellum; MTL, media temporal lobe; AY, amygdala; ST, striatum.

### Across Subcortical-Cerebellar Systems, iFC Changes with Cortical Networks are more Consistent for MTLs and Cerebellum than Striatum and Amygdala

Intrinsic connectivity changes between cerebellum and MTL respectively, and cortical networks were highly consistent in patients with accuracies of about 90% (Table 3, Figure 3). This result is well in line with previous findings reporting aberrant seed-based iFC of hippocampus and cerebellum, respectively, with cortical areas (Collin et al., 2011; Kraguljac et al., 2014). In a longitudinal study over 2 years in more than 60 patients with schizophrenia, Duan et al. (2015) demonstrated lasting iFC decrease between hippocampus and both, temporal and parietal areas, suggesting aberrant hippocampus-cortex iFC as a permanent feature in schizophrenia. Collin et al. (2011) showed consistently decreased cerebellar iFC into widespread parts of the cortex, in patients and their unaffected siblings, suggesting impaired cerebellum iFC as putative risk endophenotype for schizophrenia and therefore, as stable feature in the disease. In line with the studies from Collin et al. (2011) and Duan et al. (2015), which suggest longer lasting MTL- and cerebellum-iFC changes, we found highly consistent changes of both MTL- and cerebellum-cortex iFC in patients. This finding suggests that aberrant cortex-cerebellum iFC and cortex-MTL iFC together are stable features in schizophrenia.

In contrast, iFC between striatum and amygdala, respectively, and cortical networks separated patients from controls with significantly lower accuracy levels of about 65% (Table 3 and S3, Figure 3). Aberrant amygdala and striatum iFC with the cortex is in line with several previous studies. Anticevic et al. (2012) showed decreased iFC between amygdala and the orbitofrontal cortex, which was only present in patients of early and chronic disease stages but not in unaffected high-risk persons. This finding suggests that iFC between cortex and amygdala is modulated by disease state. Concerning striatum, previous studies demonstrated a complex pattern of in- and decreased iFC with the cortex along the ventromedial to dorsolateral axis of the striatum; in particular, this pattern of change seems to be modulated by the disease state and to be intimately linked with psychotic symptoms (Fornito et al., 2013; Manoliu et al., 2013; Peters et al., in press). However, psychotic symptoms seem to be not necessarily linked with pathophysiological striatum changes. For example, Demjaha et al. (2012) demonstrated in psychotic non-responders of anti-dopaminergic treatment, that presynaptic dopamine synthesis levels are unchanged in comparison to healthy controls, suggesting striatal dopamine synthesis being independent from psychosis. In line with the studies from Anticevic and Demjaha, which suggest inconsistent amygdala and striatum changes in patients with schizophrenia, we found that changes of both amygdala- and striatum-cortex iFC were of moderate accuracy and sensitivity, and of significant lower accuracy than iFC changes of cerebellum and MTL. Considering that cerebellum and MTL iFC changes appear as rather permanent features in schizophrenia, this finding suggests that aberrant cortex-amygdala iFC and cortex-striatum iFC are less consistent in schizophrenia and may reflect different disease states or heterogeneity across patients.

### Differential Patterns of Aberrant iFC Between Subcortical-Cerebellar Systems and Cortical Networks as Candidates to Separate Sub-Groups of Patients and/or Disease States

Widespread brain dysconnectivity is central to schizophrenia (Stephan et al., 2009). Several models proposed specific patterns of dysconnectivity being associated with specific syndromes, symptoms, states, and subgroups of patients in the disease (Aleman and Kahn, 2005; Andreasen and Pierson, 2008; Stephan et al., 2009; Tamminga et al., 2010; Williamson and Allman, 2012). Due to both their essential role in modulating the cortico-thalamic system (Buzsaki, 2006) and the overwhelming evidence of their multi-level changes in schizophrenia (Andreasen and Pierson, 2008; Benes, 2010; Tamminga et al., 2010; Howes et al., 2012), striatum, cerebellum, MTL, and amygdala are promising candidates to specify schizophrenia’s dysconnectivity. The current study provides initial evidence that these main modulatory systems of the cortico-thalamic system and their iFC with cortical networks might specify distinct dysconnectivity patterns across patients despite limitations such as medication effects in particular as laid out further below. Future studies are necessary to identify how patterns of subcortical-cerebellar systems’ dysconnectivity vary across disease states, unaffected at-risk persons, and patients, and whether specific patterns may define patient subgroups.

## Limitations and Methodological Issues

(i) Most patients of our study were treated with antipsychotic drugs (Table S1). Antipsychotic medication has been demonstrated to alter functional connectivity in patients with schizophrenia (Sambataro et al., 2010). Therefore, we cannot exclude that observed changes in iFC in comparison to healthy controls are confounded by medication effects. However, one should note that the main focus of the study was differential iFC changes across subcortical-cerebellar systems in patients i.e., differences in changes of patients instead of differences in patients relative to controls, suggesting that—due to consistent confound across patients—findings might be more robust against medication influences. Nevertheless, studies in non-medicated patients would be favorable. (ii) Sample size of the study is small, limiting the power of our study results to some degree. Therefore and due to confounding medication effects, we have to categorize our study as a preliminary study about differential iFC changes across subcortical-cerebellar systems in schizophrenia. (iii) One might ask why we used SVM instead of canonical two-sample *t*-tests to compare iFC across subjects. Typical two-sample *t*-tests for seed-based iFC maps would provide information about spatially specific group differences in cortical iFC for distinct subcortical-cerebellar systems; but such group difference maps are difficult to compare across subjects due to both, varying spatial extent of cortical changes and most importantly, the lack of direct measures about how consistent iFC changes are across systems and subjects. Combining measures of classification and their statistical comparison via binominal testing yields exactly such information. (iv) Why did we select intrinsic networks as proxies for cortico-thalamic sub-systems instead of brain-atlas-based anatomically defined regions? Concerning brain activity, we focused on slowly fluctuating activity as measured by rs-fMRI and correspondingly on iFC of such ongoing activity. Overwhelming evidence indicates that iFC is organized by intrinsic networks particularly in the human cortex and exceeds anatomically defined cortex parcellations (Fox and Raichle, 2007).

## Conclusion

Results provide preliminary evidence for more consistent iFC changes with cortical networks for MTL and cerebellum than for striatum and amygdala in schizophrenia. Differential iFC changes might reflect distinct disease states or patient subgroups.

## Author Contributions

CS and HP designed the study; JB, MS, DS and HF recruited participants and acquired data; VR, CZ and AW acquired data; JS and HP analyzed data; CS, KK, VR, AW and HP interpreted data; CS, KK and HP drafted the article; all authors critically revised and approved the final version of the article.

## Conflict of Interest Statement

The authors declare that the research was conducted in the absence of any commercial or financial relationships that could be construed as a potential conflict of interest.

## Footnotes

^1^http://www.fz-juelich.de/inb/inb-3/spm_anatomy_toolbox

^2^http://marsbar.sourceforge.net

^3^www.mathworks.com

^4^http://icatb.sourceforge.net

^5^http://www.cs.waikato.ac.nz/ml/weka/index.html
