# Supplementary material for: More Consistently Altered Connectivity Patterns for Cerebellum and Medial Temporal Lobes than for Amygdala and Striatum in Schizophrenia
Source: Front Hum Neurosci. 2016 Feb 17;10:55. doi: 10.3389/fnhum.2016.00055 (PMC4756145; doi:10.3389/fnhum.2016.00055)
Supplement: Supplementary file 1 [file Data_Sheet_1.docx]

Supplementary Material

**More Consistently Altered Connectivity Patterns for Cerebellum and Medial Temporal Lobes than for Amygdala and Striatum in Schizophrenia**

Henning Peters^1,4*^, Junming Shao^5,6,7^, Martin Scherr^1^, Dirk Schwerthöffer^1^, Claus Zimmer^2^, Hans Förstl^1^, Josef Bäuml^1^, Afra Wohlschläger^2,4^, Valentin Riedl^2,3,4^, Kathrin Koch^2,4#^, Christian Sorg^1,2,4#^

# Supplementary figures:


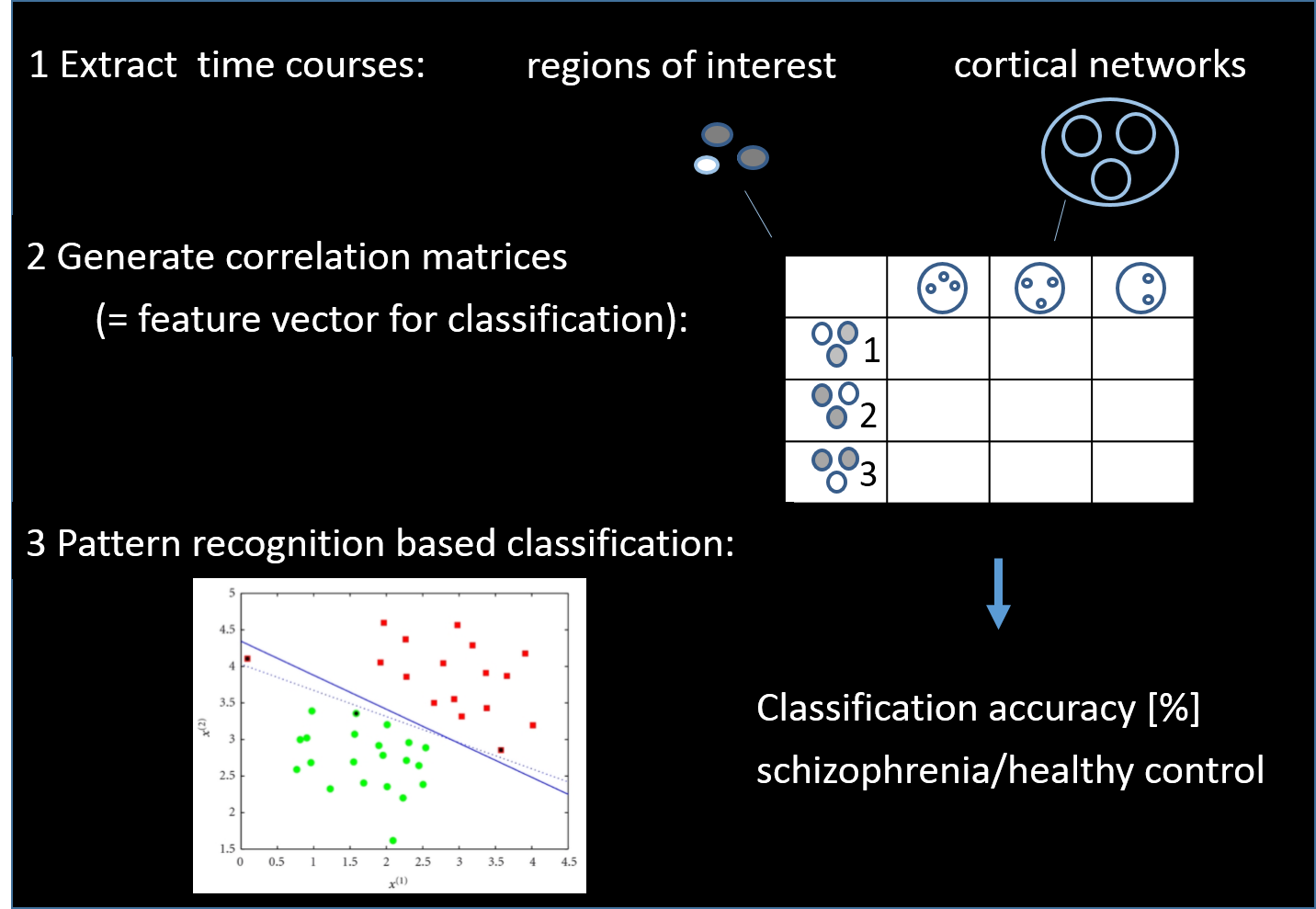


Figure S1: Data analysis pipeline. The figure illustrates the separate steps of data analysis. First, characteristic time courses of both regions-of-interest (ROIs) representing different modulatory systems and intrinsic connectivity networks representing cortico-thalamic systems were defined. Second, intrinsic functional connectivity (iFC) among ROIs and NWs time courses via Pearson’s correlation was calculated and organized in connectivity matrices. Finally, connectivity matrices were classified via support vector machine algorithm and leave-one-out-cross validation. Classification accuracies were main outputs of the whole procedure reflecting consistency of changed iFC across patients.

*
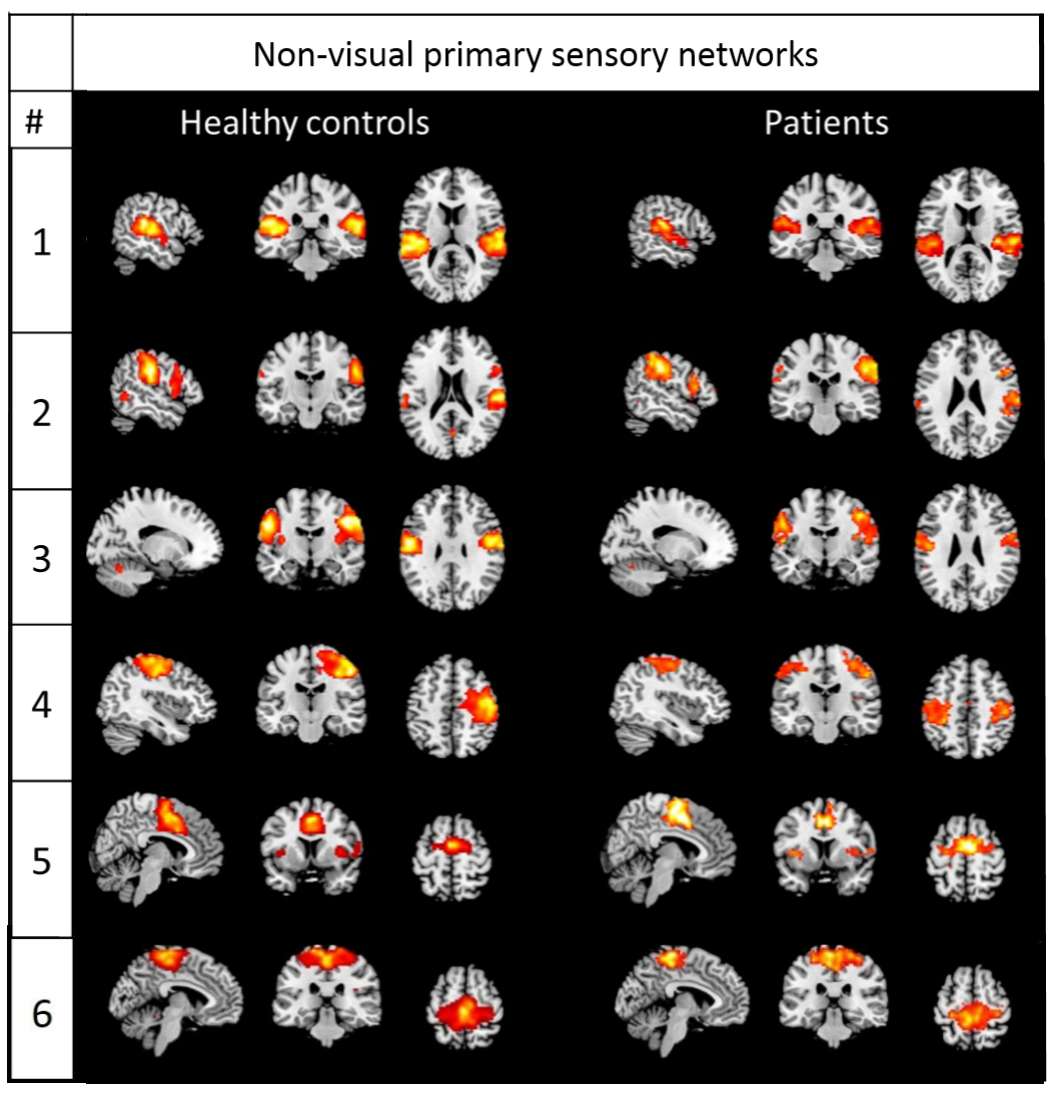
*

Figure S2: Non-visual primary sensory networks. Following the approach of Allen et al. (2011), high-model order independent component analysis (ICA) of resting state fMRI data of patients and healthy controls was performed and cortical networks of interest were selected based on templates of Allen and spatial regression analysis. This figure shows corresponding non-visual primary sensory networks for each group (one-sample t-test, p<0.05 FWE corrected, overlay on T1-weighted structural MRI). Black numbers in the left column indicate the order of ICs as used for further analysis steps.


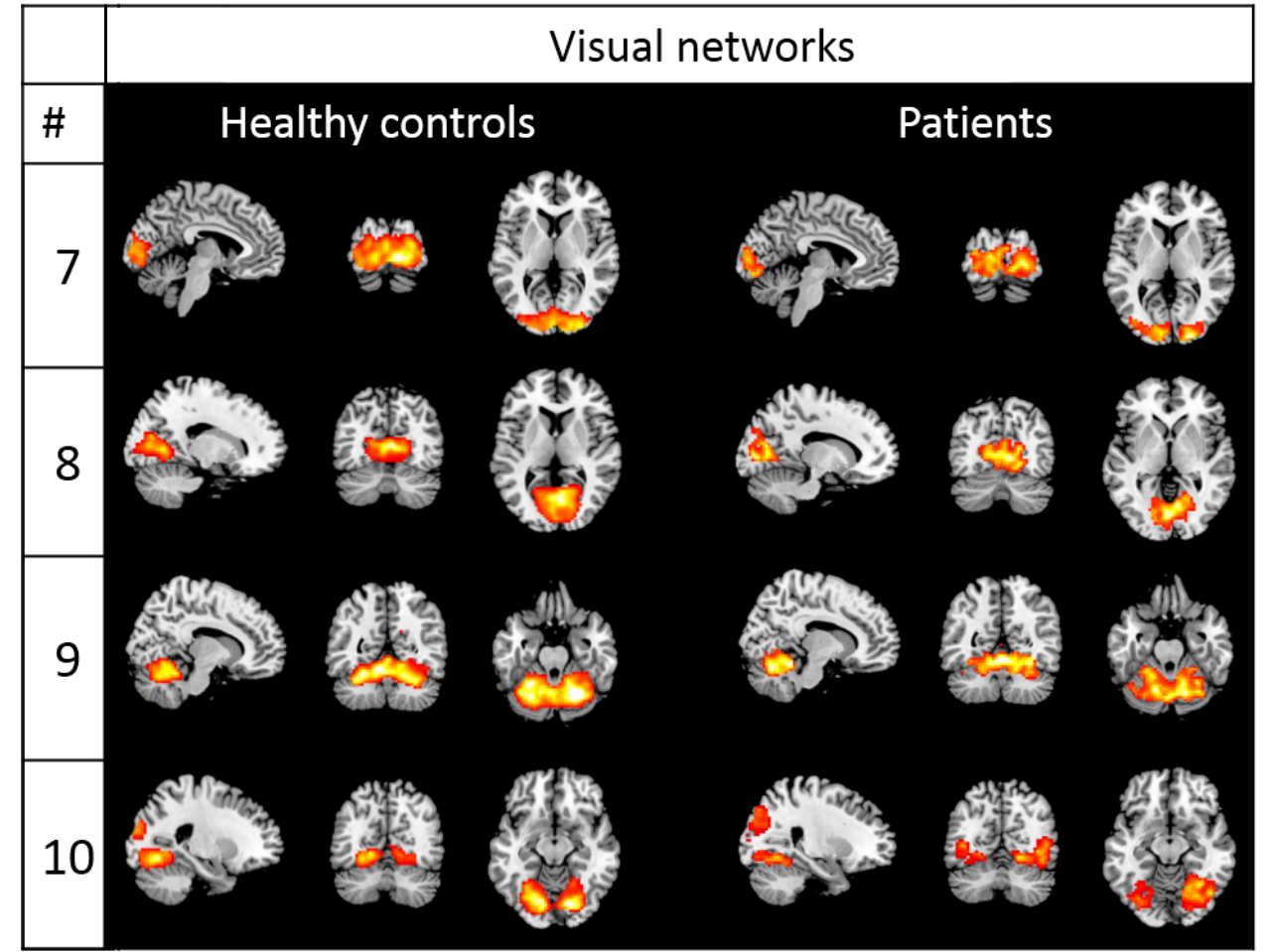


Figure S3: Visual networks. Following the approach of Allen et al. (2011), high-model order independent component analysis (ICA) of resting state fMRI data of patients and healthy controls was performed and cortical networks of interest were selected based on templates of Allen and spatial regression analysis. This figure shows corresponding visual networks for each group (one-sample t-test, p<0.05 FWE corrected, overlay on T1-weighted structural MRI). Black numbers in the left column indicate the order of ICs as used for further analysis steps.


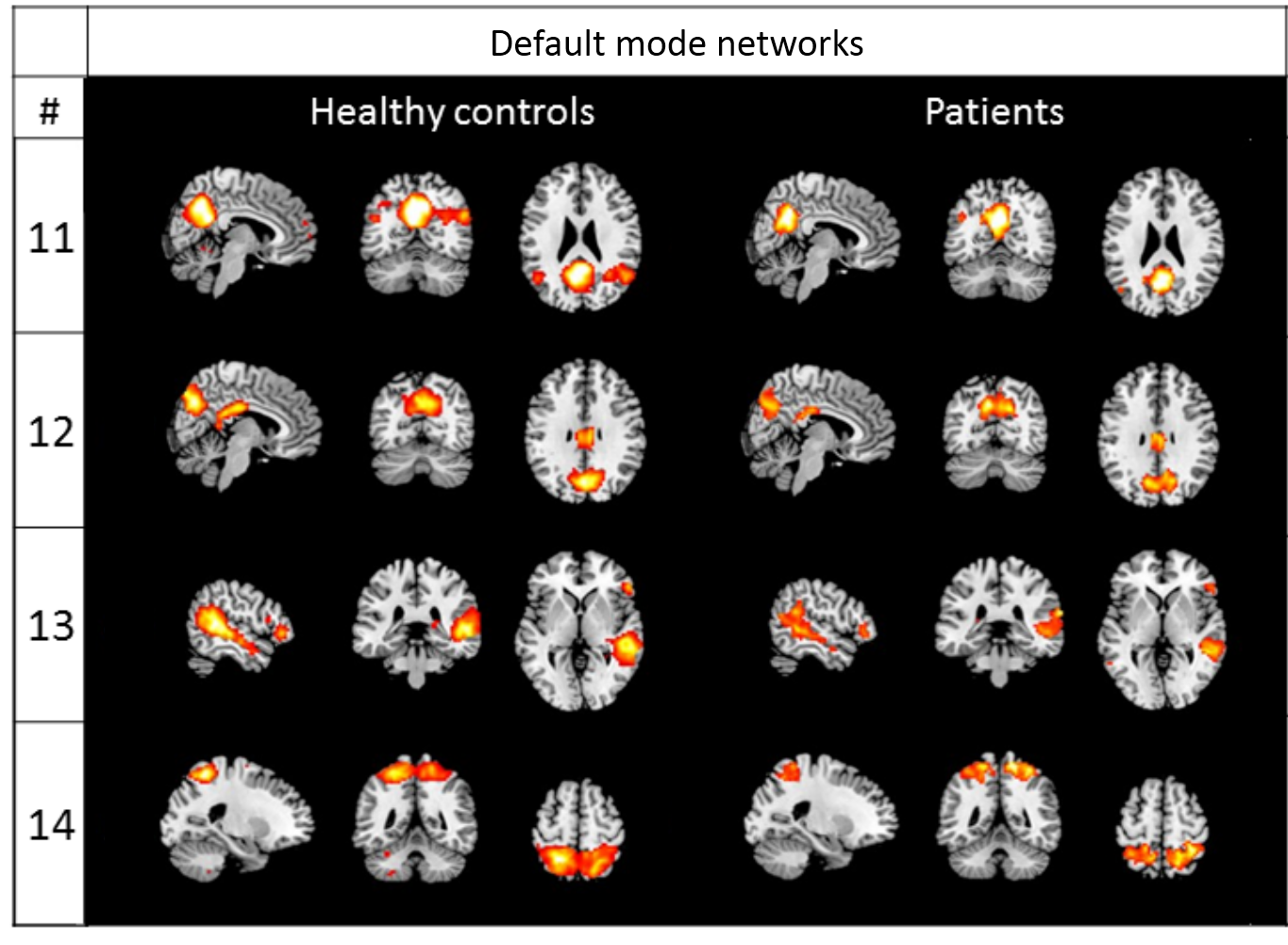


Figure S4: Default mode networks. Following the approach of Allen et al. (2011), high-model order independent component analysis (ICA) of resting state fMRI data of patients and healthy controls was performed and cortical networks of interest were selected based on templates of Allen and spatial regression analysis. This figure shows corresponding default mode networks for each group (one-sample t-test, p<0.05 FWE corrected, overlay on T1-weighted structural MRI). Black numbers in the left column indicate the order of ICs as used for further analysis steps.


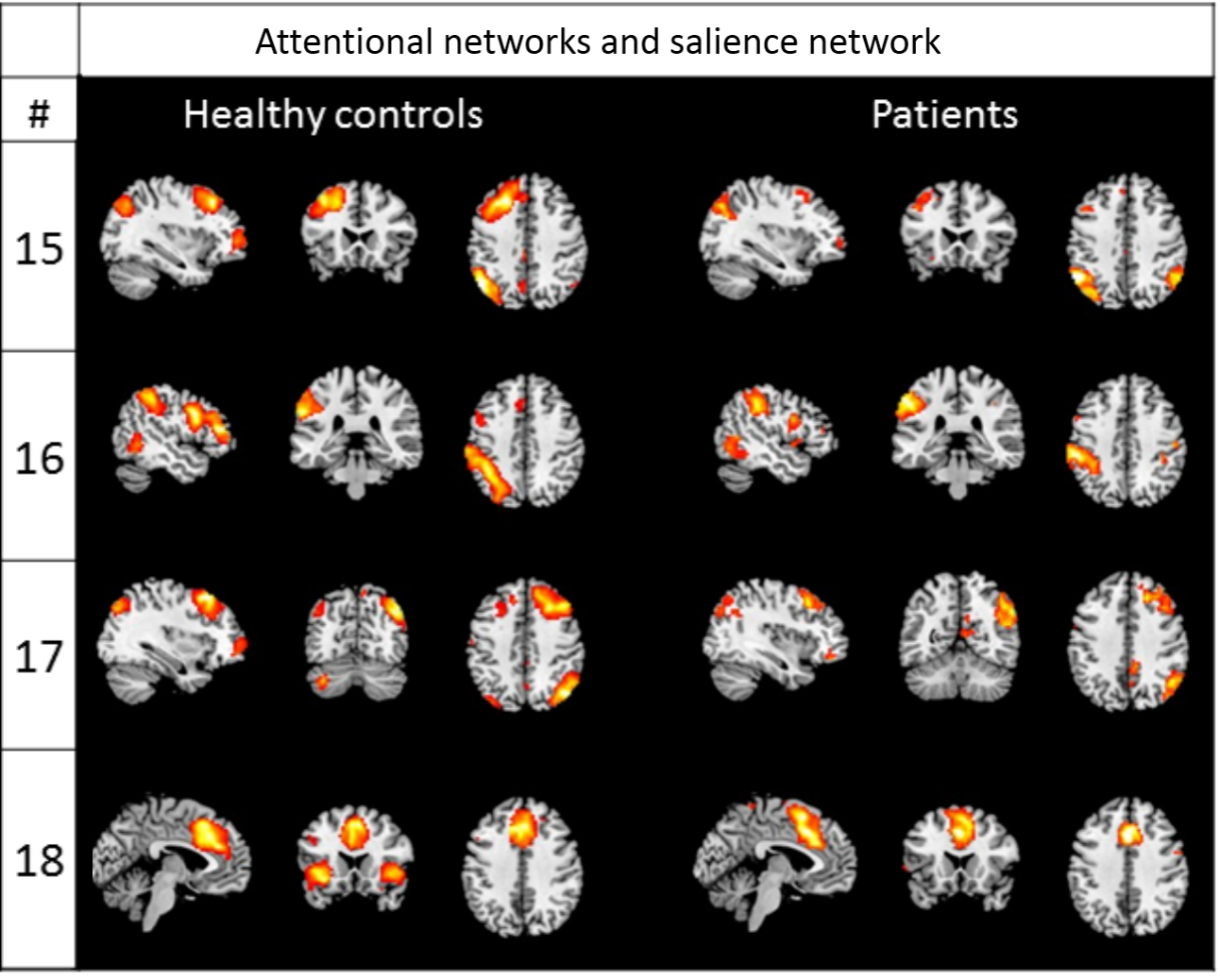


Figure S5: Attentional networks and salience network. Following the approach of Allen et al. (2011), high-model order independent component analysis (ICA) of resting state fMRI data of patients and healthy controls was performed and cortical networks of interest were selected based on templates of Allen and spatial regression analysis. This figure shows corresponding attentional and salience networks for each group (one-sample t-test, p<0.05 FWE corrected, overlay on T1-weighted structural MRI). Black numbers in the left column indicate the order of ICs as used for further analysis steps.


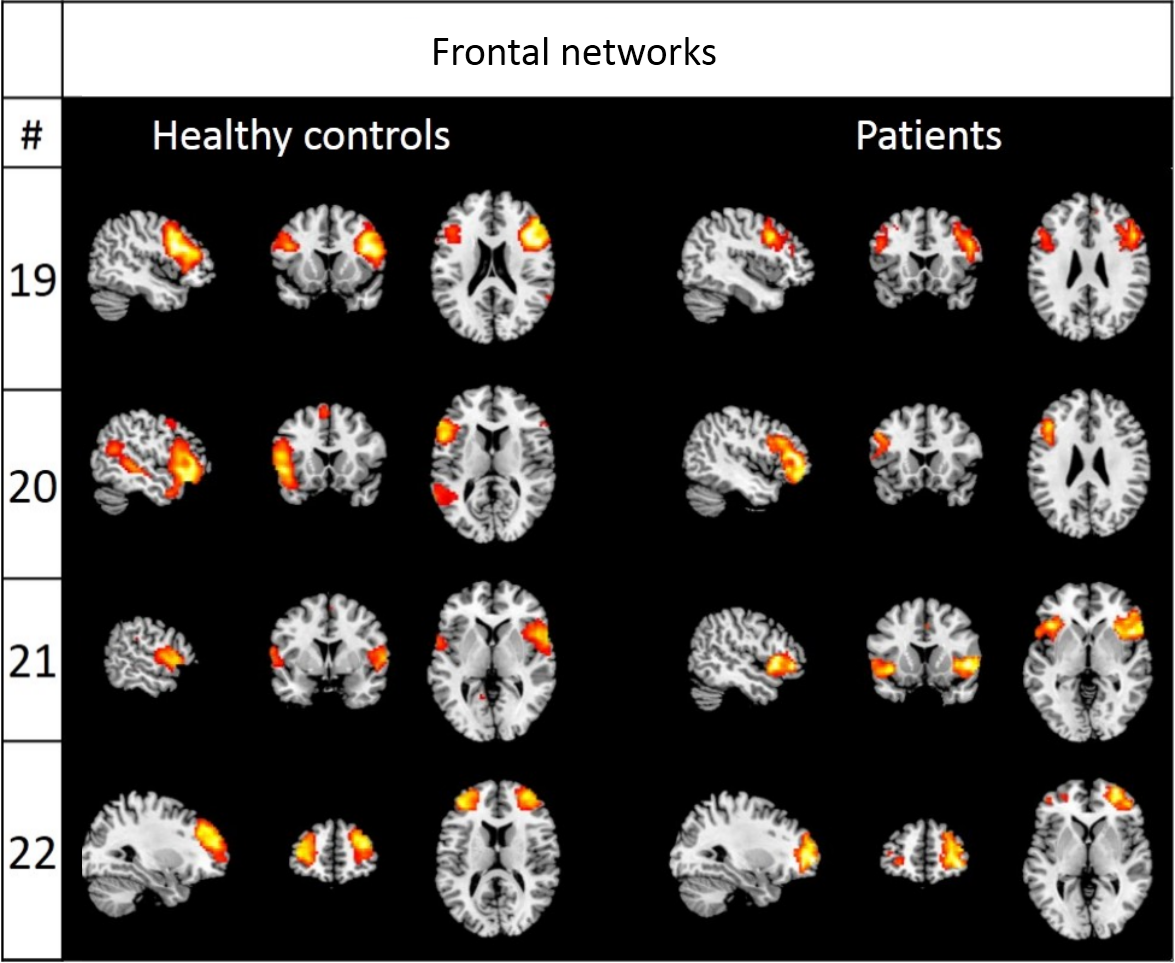


Figure S6: Frontal networks. Following the approach of Allen et al. (2011), high-model order independent component analysis (ICA) of resting state fMRI data of patients and healthy controls was performed and cortical networks of interest were selected based on templates of Allen and spatial regression analysis. This figure shows corresponding frontal networks for each group (one-sample t-test, p<0.05 FWE corrected, overlay on T1-weighted structural MRI). Black numbers in the left column indicate the order of ICs as used for further analysis steps.

# Supplementary tables:

## **Table S1**: Patients’ individual medication

| **Patient_ID** | **Medication** |
| --- | --- |
| 1 | 20 mg Olanzapine |
| 2 | 100 mg Clozapine, 80 mg Ziprasidone |
| 3 | 30 mg Olanzapine, 15 mg Aripiprazole |
| 4 | 10 mg Olanzapine, 5 mg Risperidone |
| 5 | 30 mg Olanzapine, 5 mg Risperidone |
| 6 | *NO medication* |
| 7 | 12.5 mg Olanzapine, 6 mg Paliperidone |
| 8 | *NO medication* |
| 9 | 20 mg Olanzapine |
| 10 | 400 mg Quetiapine, 9 mg Paliperidone |
| 11 | 30 mg Olanzapine, 50 mg Clozapine |
| 12 | 30 mg Olanzapine, 5 mg Risperidone |
| 13 | 400 mg Quetiapine, 5 mg Risperidone |
| 14 | 25 mg Olanzapine, 50 mg Clozapine |
| 15 | 200 mg Amisulpride, 15 mg Aripiprazole |
| 16 | 30 mg Olanzapine, 400 mg Quetiapine |
| 17 | 15 mg Olanzapine |
| 18 | 200 mg Clozapine, 12mg Paliperidone |

# References

Allen, E.A., Erhardt, E.B., Damaraju, E., Gruner, W., Segall, J.M., Silva, R.F., Havlicek, M., Rachakonda, S., Fries, J., Kalyanam, R., Michael, A.M., Caprihan, A., Turner, J.A., Eichele, T., Adelsheim, S., Bryan, A.D., Bustillo, J., Clark, V.P., Feldstein Ewing, S.W., Filbey, F., Ford, C.C., Hutchison, K., Jung, R.E., Kiehl, K.A., Kodituwakku, P., Komesu, Y.M., Mayer, A.R., Pearlson, G.D., Phillips, J.P., Sadek, J.R., Stevens, M., Teuscher, U., Thoma, R.J., Calhoun, V.D. (2011) A baseline for the multivariate comparison of resting-state networks. Front Syst Neurosci, 5:2.

Manoliu, A., Riedl, V., Zherdin, A., Muhlau, M., Schwerthoffer, D., Scherr, M., Peters, H., Zimmer, C., Forstl, H., Bauml, J., Wohlschlager, A.M., Sorg, C. (2014) Aberrant dependence of default mode/central executive network interactions on anterior insular salience network activity in schizophrenia. Schizophr Bull, 40:428-37.
